# Supplementary material for: Associations between Vertebral Localized Contrast Changes and Adjacent Annular Fissures in Patients with Low Back Pain: A Radiomics Approach
Source: J Clin Med. 2023 Jul 25;12(15):4891. doi: 10.3390/jcm12154891 (PMC10420134; doi:10.3390/jcm12154891)
Supplement: Supplementary file 1 [file jcm-12-04891-s001.zip › jcm-2519291-supplementary.pdf]

Table S1. ICC scores

Feature name	ICC(1,1) Reference ROI vs ROI eroded by one pixel	ICC(1,1) Reference image (1x1x1 mm^3) vs image with increased voxel size (1.1x1.1x1.1 mm^3)
firstorder 10Percentile [t1w]	0.952036987	0.997827572
firstorder 90Percentile [t1w]	0.999823728	0.999939236
firstorder Energy [t1w]	0.931081344	0.506974506
firstorder Entropy [t1w]	0.944959433	0.998428214
firstorder InterquartileRange [t1w]	0.963752516	0.998577311
firstorder Kurtosis [t1w]	0.931856065	0.981082968
firstorder Maximum [t1w]	0.993901721	0.992059858
firstorder MeanAbsoluteDeviation [t1w]	0.942121374	0.998181942
firstorder Mean [t1w]	0.995605411	0.999841969
firstorder Median [t1w]	0.998143337	0.999913803
firstorder Minimum [t1w]	0.424895481	0.822918549
firstorder Range [t1w]	0.943960185	0.982725791
firstorder RobustMeanAbsoluteDeviation	0.958040868	0.998505908
firstorder RootMeanSquared [t1w]	0.996141583	0.999859683
firstorder Skewness [t1w]	0.821714229	0.990138844
firstorder TotalEnergy [t1w]	0.931081344	0.996806424
firstorder Uniformity [t1w]	0.955462258	0.998585638
firstorder Variance [t1w]	0.929586512	0.997845749
glcm Autocorrelation [t1w]	0.845427903	0.959180218
glcm ClusterProminence [t1w]	0.944135579	0.996031567
glcm ClusterShade [t1w]	0.922093591	0.992257732
glcm ClusterTendency [t1w]	0.952379985	0.997961009
glcm Contrast [t1w]	0.859250886	0.976670984
glcm Correlation [t1w]	0.947404452	0.913490049
glcm DifferenceAverage [t1w]	0.927892477	0.982117253
glcm DifferenceEntropy [t1w]	0.912121822	0.981813265
glcm DifferenceVariance [t1w]	0.766253767	0.970400565
glcm Id [t1w]	0.970105782	0.982296892
glcm Idm [t1w]	0.973575091	0.982316615
glcm Idmn [t1w]	0.801504356	0.783385402
glcm Idn [t1w]	0.860117598	0.790320886
glcm Imc1 [t1w]	0.95907545	0.892308463
glcm Imc2 [t1w]	0.970143232	0.958152872
glcm InverseVariance [t1w]	0.96459602	0.985182537
glcm JointAverage [t1w]	0.869281547	0.966784157
glcm JointEnergy [t1w]	0.970514409	0.997149129
glcm JointEntropy [t1w]	0.951488589	0.997424467
glcm MCC [t1w]	0.949848797	0.926139498
glcm MaximumProbability [t1w]	0.98612611	0.992384579
glcm SumAverage [t1w]	0.869281547	0.966784157
glcm SumEntropy [t1w]	0.957310773	0.998222743
glcm SumSquares [t1w]	0.947487762	0.998222545
gldm DependenceEntropy [t1w]	0.905536355	0.94573212
gldm DependenceNonUniformity [t1w]	0.921272716	0.669388459
gldm DependenceNonUniformityNormalized	0.997775678	0.969001701
gldm DependenceVariance [t1w]	0.998761535	0.962864439
gldm GrayLevelNonUniformity [t1w]	0.99227742	0.710842449
gldm GrayLevelVariance [t1w]	0.929593663	0.997826896
gldm HighGrayLevelEmphasis [t1w]	0.848980526	0.959115438
gldm LargeDependenceEmphasis [t1w]	0.990296312	0.96814774
gldm LargeDependenceHighGrayLevelEmph	0.656229761	0.827587708

gldm_LargeDependenceLowGrayLevelEmph	0.865770456	0.97889159
gldm_LowGrayLevelEmphasis [t1w]	0.808851754	0.901610522
gldm_SmallDependenceEmphasis [t1w]	0.947722069	0.956624338
gldm_SmallDependenceHighGrayLevelEmph	0.879946811	0.972310788
gldm_SmallDependenceLowGrayLevelEmph	0.552009507	0.570901669
glrlm_GrayLevelNonUniformity [t1w]	0.990087535	0.671031733
glrlm_GrayLevelNonUniformityNormalized	0.945283906	0.998282795
glrlm_GrayLevelVariance [t1w]	0.924865197	0.997667324
glrlm_HighGrayLevelRunEmphasis [t1w]	0.851560742	0.959197125
glrlm_LongRunEmphasis [t1w]	0.988701031	0.949790203
glrlm_LongRunHighGrayLevelEmphasis [t1w]	0.808602252	0.937679963
glrlm_LongRunLowGrayLevelEmphasis [t1w]	0.851208755	0.947753875
glrlm_LowGrayLevelRunEmphasis [t1w]	0.810374162	0.89737384
glrlm_RunEntropy [t1w]	0.918616584	0.9906617
glrlm_RunLengthNonUniformity [t1w]	0.884235964	0.538615518
glrlm_RunLengthNonUniformityNormalized	0.980838845	0.97211206
glrlm_RunPercentage [t1w]	0.985291649	0.968675711
glrlm_RunVariance [t1w]	0.992352594	0.936665242
glrlm_ShortRunEmphasis [t1w]	0.980996745	0.970274815
glrlm_ShortRunHighGrayLevelEmphasis [t1w]	0.857967095	0.961757064
glrlm_ShortRunLowGrayLevelEmphasis [t1w]	0.797655807	0.881669802
glszm_GrayLevelNonUniformity [t1w]	0.845652175	0.771972635
glszm_GrayLevelNonUniformityNormalized	0.930681289	0.991530566
glszm_GrayLevelVariance [t1w]	0.919594004	0.995342892
glszm_HighGrayLevelZoneEmphasis [t1w]	0.893306508	0.961709721
glszm_LargeAreaEmphasis [t1w]	0.951548813	0.848300631
glszm_LargeAreaHighGrayLevelEmphasis	0.945968229	0.708636125
glszm_LargeAreaLowGrayLevelEmphasis	0.810655497	0.889590957
glszm_LowGrayLevelZoneEmphasis [t1w]	0.770724043	0.860216918
glszm_SizeZoneNonUniformity [t1w]	0.827131723	0.940835587
glszm_SizeZoneNonUniformityNormalized	0.791671906	0.779462071
glszm_SmallAreaEmphasis [t1w]	0.812242869	0.775829287
glszm_SmallAreaHighGrayLevelEmphasis	0.88603705	0.963640516
glszm_SmallAreaLowGrayLevelEmphasis	0.722943331	0.79999493
glszm_ZoneEntropy [t1w]	0.958432333	0.924058971
glszm_ZonePercentage [t1w]	0.944997976	0.95764563
glszm_ZoneVariance [t1w]	0.953096273	0.847093751
ngtdm_Busyness [t1w]	0.921298181	0.90062348
ngtdm_Coarseness [t1w]	0.9411828	0.617299181
ngtdm_Complexity [t1w]	0.922403429	0.987942391
ngtdm_Contrast [t1w]	0.831988775	0.932074682
ngtdm_Strength [t1w]	0.97061072	0.938778064
firstorder_10Percentile [t2w]	0.943533904	0.997270745
firstorder_90Percentile [t2w]	0.99973375	0.999896646
firstorder_Energy [t2w]	0.925238582	0.471918985
firstorder_Entropy [t2w]	0.942092967	0.997816192
firstorder_InterquartileRange [t2w]	0.972513067	0.998365862
firstorder_Kurtosis [t2w]	0.912850115	0.975677388
firstorder_Maximum [t2w]	0.975649573	0.982775578
firstorder_MeanAbsoluteDeviation [t2w]	0.946769722	0.998019797
firstorder_Mean [t2w]	0.994612652	0.999794459
firstorder_Median [t2w]	0.997906244	0.999875482
firstorder_Minimum [t2w]	0.124766472	0.734336301
firstorder_Range [t2w]	0.929433629	0.978134459
firstorder_RobustMeanAbsoluteDeviation	0.967266125	0.998545212
firstorder_RootMeanSquared [t2w]	0.99537219	0.999821527
firstorder_Skewness [t2w]	0.767509039	0.983040008
firstorder_TotalEnergy [t2w]	0.925238582	0.996682988
firstorder_Uniformity [t2w]	0.958260755	0.997981065
firstorder_Variance [t2w]	0.932912171	0.997621433
glcm_Autocorrelation [t2w]	0.85267657	0.971955242

glcm_ClusterProminence [t2w]	0.960767775	0.995879489
glcm_ClusterShade [t2w]	0.955932902	0.995121637
glcm_ClusterTendency [t2w]	0.962750896	0.998066276
glcm_Contrast [t2w]	0.901115255	0.98603584
glcm_Correlation [t2w]	0.961936798	0.944411565
glcm_DifferenceAverage [t2w]	0.946159318	0.9894058
glcm_DifferenceEntropy [t2w]	0.928482149	0.987395417
glcm_DifferenceVariance [t2w]	0.823367687	0.979880925
glcm_Id [t2w]	0.976296775	0.987691308
glcm_Idm [t2w]	0.979454053	0.986917538
glcm_Idmn [t2w]	0.789693595	0.785588301
glcm_Idn [t2w]	0.809827917	0.797904114
glcm_Imc1 [t2w]	0.97414292	0.953176381
glcm_Imc2 [t2w]	0.979399108	0.982634578
glcm_InverseVariance [t2w]	0.976266682	0.988795273
glcm_JointAverage [t2w]	0.866857769	0.973795629
glcm_JointEnergy [t2w]	0.974288796	0.996862336
glcm_JointEntropy [t2w]	0.955686706	0.997913116
glcm_MCC [t2w]	0.94923879	0.938685696
glcm_MaximumProbability [t2w]	0.986693848	0.992504697
glcm_SumAverage [t2w]	0.866857769	0.973795629
glcm_SumEntropy [t2w]	0.961678441	0.997948297
glcm_SumSquares [t2w]	0.957131611	0.998331142
gldm_DependenceEntropy [t2w]	0.892982554	0.953120959
gldm_DependenceNonUniformity [t2w]	0.913070854	0.649241962
gldm_DependenceNonUniformityNormalized	0.996592606	0.977297692
gldm_DependenceVariance [t2w]	0.99906154	0.966256424
gldm_GrayLevelNonUniformity [t2w]	0.99331998	0.704760261
gldm_GrayLevelVariance [t2w]	0.932936294	0.997603518
gldm_HighGrayLevelEmphasis [t2w]	0.858575831	0.972368695
gldm_LargeDependenceEmphasis [t2w]	0.991318788	0.971616525
gldm_LargeDependenceHighGrayLevelEmph	0.649186091	0.86841728
gldm_LargeDependenceLowGrayLevelEmph	0.8825577	0.965054747
gldm_LowGrayLevelEmphasis [t2w]	0.830421786	0.88397927
gldm_SmallDependenceEmphasis [t2w]	0.968198044	0.973318949
gldm_SmallDependenceHighGrayLevelEmph	0.90770512	0.983618139
gldm_SmallDependenceLowGrayLevelEmph	0.485022635	0.595785847
glrlm_GrayLevelNonUniformity [t2w]	0.992272638	0.682605154
glrlm_GrayLevelNonUniformityNormalized	0.951924429	0.997769606
glrlm_GrayLevelVariance [t2w]	0.928902572	0.997425534
glrlm_HighGrayLevelRunEmphasis [t2w]	0.860258587	0.972434573
glrlm_LongRunEmphasis [t2w]	0.989999449	0.9527398
glrlm_LongRunHighGrayLevelEmphasis [t2w]	0.827364906	0.96079628
glrlm_LongRunLowGrayLevelEmphasis [t2w]	0.866649856	0.919100515
glrlm_LowGrayLevelRunEmphasis [t2w]	0.83159931	0.880391887
glrlm_RunEntropy [t2w]	0.919709299	0.994068828
glrlm_RunLengthNonUniformity [t2w]	0.883954762	0.475944344
glrlm_RunLengthNonUniformityNormalized	0.985096661	0.97789911
glrlm_RunPercentage [t2w]	0.987758859	0.973855993
glrlm_RunVariance [t2w]	0.992493888	0.93679509
glrlm_ShortRunEmphasis [t2w]	0.985135208	0.975641316
glrlm_ShortRunHighGrayLevelEmphasis [t2w]	0.865717113	0.973947362
glrlm_ShortRunLowGrayLevelEmphasis [t2w]	0.821346647	0.868877543
glszm_GrayLevelNonUniformity [t2w]	0.958090925	0.680442829
glszm_GrayLevelNonUniformityNormalized	0.867144834	0.988981882
glszm_GrayLevelVariance [t2w]	0.878929548	0.993805254
glszm_HighGrayLevelZoneEmphasis [t2w]	0.888623456	0.974020073
glszm_LargeAreaEmphasis [t2w]	0.98011957	0.880485477
glszm_LargeAreaHighGrayLevelEmphasis	0.957059533	0.811233477
glszm_LargeAreaLowGrayLevelEmphasis	0.875866652	0.885879933
glszm_LowGrayLevelZoneEmphasis [t2w]	0.806077479	0.849453734

glszm SizeZoneNonUniformity [t2w]	0.86570541	0.887481911
glszm SizeZoneNonUniformityNormalized	0.787862426	0.852233141
glszm SmallAreaEmphasis [t2w]	0.793181801	0.843005747
glszm SmallAreaHighGrayLevelEmphasis	0.893594725	0.976570245
glszm SmallAreaLowGrayLevelEmphasis	0.740845813	0.811980289
glszm ZoneEntropy [t2w]	0.937407687	0.933730469
glszm ZonePercentage [t2w]	0.973299501	0.975326973
glszm ZoneVariance [t2w]	0.980659475	0.879500587
ngtdm Busyness [t2w]	0.931342616	0.897389547
ngtdm Coarseness [t2w]	0.934536328	0.602570788
ngtdm Complexity [t2w]	0.921956169	0.991556562
ngtdm Contrast [t2w]	0.861854114	0.936111242
ngtdm Strength [t2w]	0.974017452	0.948797955

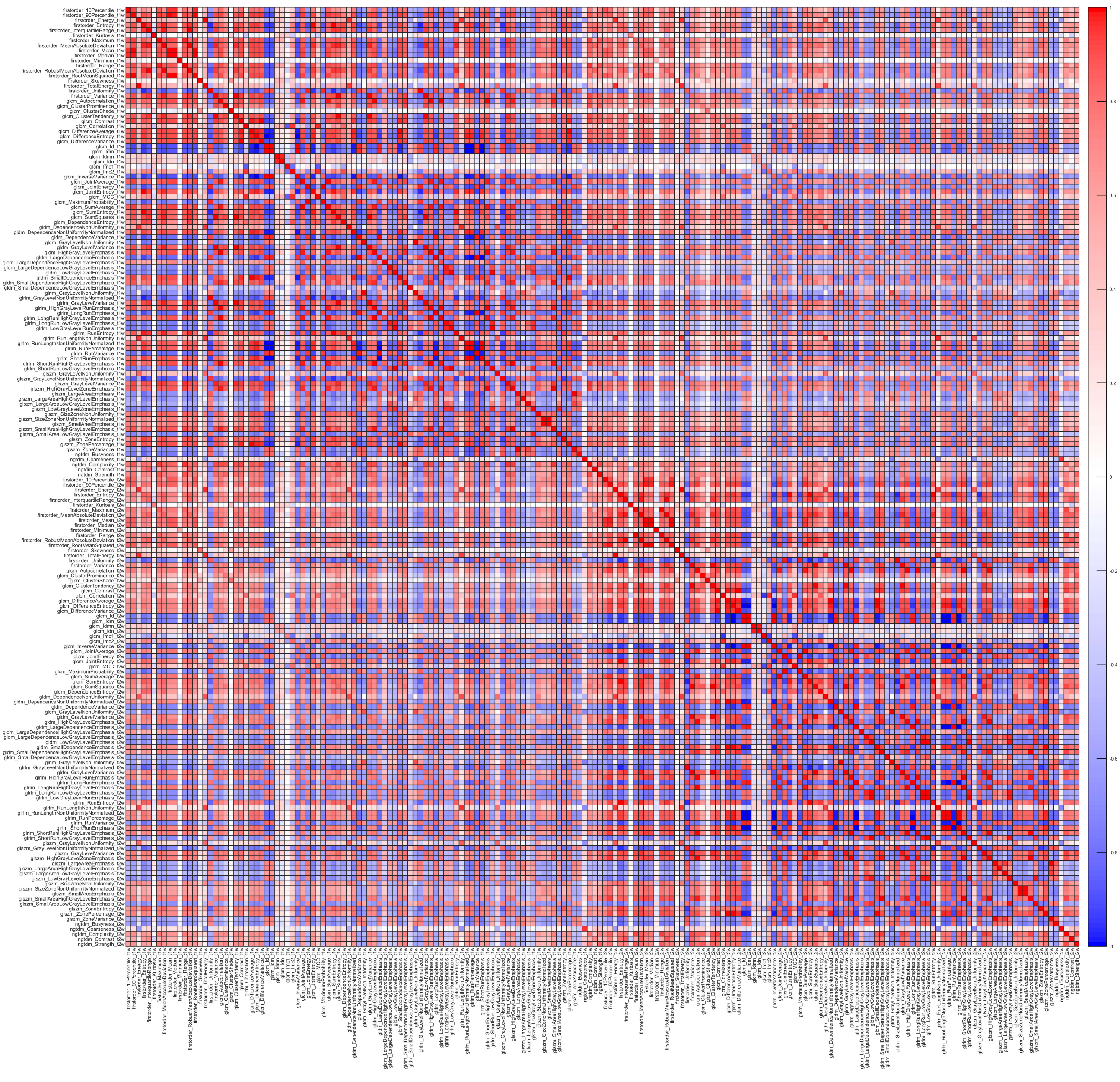

Figure S1. Feature correlation matrix.
